# Development of a novel sandwich immunoassay based on targeting recombinant Francisella outer membrane protein A for the diagnosis of tularemia

**DOI:** 10.3389/fcimb.2024.1455259

**Published:** 2024-08-20

**Authors:** Jieun Jang, Do Hyung Kwon, Ju-Hong Jang, Dong-Gwang Lee, Seo-Hyuk Chang, Min-Young Jeon, Young-Su Jeong, Dong-Hyun Song, Jeong-Ki Min, Jong-Gil Park, Moo-Seung Lee, Baek-Soo Han, Wonjun Yang, Nam-Kyung Lee, Jangwook Lee

**Affiliations:** ^1^ Biotherapeutics Translational Research Center, Korea Research Institute of Bioscience and Biotechnology, Daejeon, Republic of Korea; ^2^ Department of Biomolecular Science, Korea Research Institute of Bioscience and Biotechnology, School of Bioscience, Korea University of Science and Technology, Daejeon, Republic of Korea; ^3^ Chem-Bio Technology Center, Agency for Defense Development, Daejeon, Republic of Korea; ^4^ Environmental Diseases Research Center, Korea Research Institute of Bioscience and Biotechnology, Daejeon, Republic of Korea; ^5^ Biodefense Research Center, Korea Research Institute of Bioscience and Biotechnology, Daejeon, Republic of Korea

**Keywords:** tularemia, *Francisella tularensis*, FopA, tier 1 select agent, sandwich immunoassay

## Abstract

**Introduction:**

Tularemia, caused by the bacterium *Francisella tularensis*, poses health risks to humans and can spread through a variety of routes. It has also been classified as a Tier 1 Select agent by the CDC, highlighting its potential as a bioterrorism agent. Moreover, it is difficult to diagnose in a timely fashion, owing to the non-specific nature of tularemia infections. Rapid, sensitive, and accurate detection methods are required to reduce mortality rates. We aimed to develop antibodies directed against the outer membrane protein A of *F. tularensis* (FopA) for rapid and accurate diagnosis of tularemia.

**Methods:**

We used a baculovirus insect cell expression vector system to produce the FopA antigen and generate anti-FopA antibodies through immunization of BALB/c mice. We then employed hybridoma and phage display technologies to screen for antibodies that could recognize unique epitopes on FopA.

**Result:**

Two monoclonal antibodies, 6B12 and 3C1, identified through phage display screening specifically bound to recombinant FopA in a dose-dependent manner. The binding affinity of the anti-FopA 6B12 and 3C1 antibodies was observed to have an equilibrium dissociation constant of 1.76 × 10-10 M and 1.32 × 10-9 M, respectively. These antibodies were used to develop a sandwich ELISA system for the diagnosis of tularemia. This assay was found to be highly specific and sensitive, with detection limits ranging from 0.062 ng/mL in PBS to 0.064 ng/mL in skim milk matrices.

**Discussion:**

Our findings demonstrate the feasibility of a novel diagnostic approach for detecting *F. tularensis* based on targeting FopA, as opposed to existing tests that target the bacterial lipopolysaccharide.

## 1 Introduction

Tularemia is a disease that affects humans and animals and is caused by the bacterium *Francisella tularensis* (*F. tularensis*) (Çelebi et al., 2006). Infection can occur through direct contact with infected animals or their tissues, bites from infected ticks or flies, or exposure to contaminated water or soil (Hickey et al., 2011; Sharma et al., 2023). According to the U.S. Centers for Disease Control and Prevention (CDC), approximately 200 cases of human infections are reported annually in the United States, with a consistently increasing trend having occurred (Bishop et al., 2023). The disease is widespread in the Northern Hemisphere and is found in a few countries in the Southern Hemisphere. Although it is generally considered to be a rare disease, it is frequently found in Northern and Central Europe and in countries of the former Soviet Union, which have reported the highest number of human cases (Hestvik et al., 2015; Lindgren et al., 2024). The disease exhibits significant annual and seasonal variation, with most outbreaks being local and sporadic but mostly occurring during late summer and autumn (Maurin and Gyuranecz, 2016). Research on tularemia has gained increased attention over the past two decades because of the classification of *F. tularensis* as a Tier 1 Select agent by the CDC, which highlights its high morbidity and mortality, ease of aerosolization, and low infectious dose (Genchi et al., 2015; Nelson and Sjöstedt, 2024). *F. tularensis* poses a significant threat as a Category A potential agent of bioterrorism, along with *Bacillus anthracis*, *Yersinia pestis*, smallpox virus, and botulinum neurotoxin (Altman and Wachs, 2002; Lamps et al., 2004).

The clinical manifestations of tularemia often make clinical diagnosis challenging owing to its non-specific nature, which frequently mimics that of influenza or other respiratory infections (Lamps et al., 2004). In some cases, patients develop systemic illnesses. Inhalation of infectious aerosols can lead to severe pneumonia, with mortality rates as high as 60% (Boisset et al., 2014; Zellner and Huntley, 2019). The lack of available vaccines and the limited effectiveness of a small group of antibiotics in treating tularemia further underscore the importance of administering appropriate treatment in the early stages of the disease (Maurin et al., 2024). Although efficient antibiotic therapy is available, delayed diagnosis can result in increased mortality rates. Therefore, rapid, sensitive, and accurate detection methods are required (Lamps et al., 2004).

Various diagnostic methods for *F. tularensis* infection in humans and animals, including serological and molecular biological procedures, are available (Yang and Rothman, 2004; Walker, 2014). Although bacterial culture-based diagnosis is considered the gold standard, it is time-consuming, laborious, and poses a risk of infection to operators (Wiesinger-Mayr et al., 2007; Yao et al., 2022). Molecular biological methods, such as real-time polymerase chain reaction (RT-PCR), are safe, reliable, highly sensitive, and specific, but are conversely time-consuming, difficult to operate, and require precise equipment (Kim et al., 2015; Liu et al., 2019). Another method for detecting *F. tularensis* infections involves immunological techniques, which employ antibodies to detect specific antigens, thereby revealing the presence of bacteria (Maurin, 2020; Hannah et al., 2021). To date, various diagnostic kits have been commercialized for sensitive and specific detection of *F. tularensis* using molecular biological and immunological techniques (**Table 1**). Despite the availability of these kits, information on their target antigens and detection ranges is lacking. Lipopolysaccharide (LPS) is the predominant outer membrane component of Gram-negative bacteria. Employing LPS as a diagnostic antigen is insufficient for differentiating between infections caused by cross-reactive species, such as *Brucella*, *Yersinia enterocolitica*, *Vibrio cholerae*, and *Escherichia coli*, which often leads to false positives (Pelletier et al., 2009; Yanes et al., 2018). Owing to the predominance of LPS in the outer membrane of bacteria, which is targeted by most commercial *F. tularensis* detection kits, cross-reactivity with undesired bacteria is possible (Yanes et al., 2018). Many studies have reported that bacterial outer membrane proteins (Omp) have strong immunogenicity and can be substituted for LPS (Ahmed et al., 2015; Nagaratnam et al., 2022). Furthermore, Omp antigens can significantly reduce false-positive results caused by cross-reactive bacteria (Yao et al., 2022). Therefore, it is important to develop new detection methods that can specifically and sensitively identify *F. tularensis* antigens rather than relying on LPS targeting.

**Table 1:** Commercial *Francisella tularensis* detection kit.

Product	Method	Company	Target	Characteristics	Reference
VIRAPID^®^ TULAREMIA	LFA^a^	Vircell	LPS^b^	99.1% sensitivity and 98.6% specificity	(Kılıç et al., 2012; Chaignat et al., 2014)
BADD Tularemia Biowarfare Detection Test Kit	LFA^a^	ADVNT biotechnologies	Not reported	1.48 x 10^6^ cfu/mL of LoD^c^	https://advnt.org
Raid™ 8	LFA^a^	Alexeter Technologies	Not reported	1.6 x 10^6^ cfu/mL of LoD^c^	https://www.alexeter.com
Tularemia BioThreat Alert^®^ Kit	LFA^a^	Tetracore	Not reported	1.0 x 10^7^~ 1 x10^8^ cfu/mL of LoD^c^	(Pillai et al., 2020)
SERION ELISA classic Francisella tularensis	ELISA	SERION diagnostics	LPS^b^	86.3% sensitivity and 95.5% specificity	(Yanes et al., 2018)
Tularemia test kit Biotoxis	RT-PCR	Bertin	Not reported	90.32~96.55% sensitivity, 1,000 cfu/L LoD/49 copies	(Hennebique et al., 2020)
Francisella tularensis RT-PCR Kit	RT-PCR	BioPerfectus	Not reported	5 copies/reaction of LoD^c^	https://www.bioperfectus.com

a Lateral Flow Assay.

b Lipopolysaccharide.

c Limit of Detection.

This study aimed to develop antibodies that can bind to the outer membrane protein A of *F. tularensis* (FopA) for diagnostic purposes. FopA is a protein consisting of 393 amino acids with less than 40% sequence identity to known bacterial outer membrane proteins, and is highly immunogenic (Nagaratnam et al., 2022). Several studies have shown that FopA functions as a protective antigen against tularemia. In this study, we utilized recombinant FopA antigen produced by an insect cell-based expression system to immunize mice and identified high-affinity and sensitive antibodies that bind to FopA through two distinct screening methods. We then assessed the limit of detection of a sandwich immunoassay established using two selected antibodies for detecting FopA in various buffer matrices. Our findings demonstrate the feasibility of a novel diagnostic approach for detecting *F. tularensis*.

## 2 Materials and methods

### 2.1 Cell lines

Expi293F™ cells were grown in suspension culture with expression medium (Gibco, A14351-02) at 37°C in a 70% humid, 5% CO_2_ incubator. For hybridoma fusion, Sp2/0-Ag14 (ATCC) cells were grown in Medium A (STEMCELL Technologies) and DMEM supplemented with 10% Fetal Bovine Serum (R&D systems), 1% Antibiotic-Antimycotic (Gibco) at 37°C in a 5% CO_2_ incubator.

### 2.2 Generation of FopA-encoding bacmid

The *fopA* gene (FTT0583) was synthesized by Gene-art (Thermo Fisher Scientific). The pFastBac-FopA donor plasmid was constructed using the pFastBac™ vector in the Bac-to-Bac™ Vector Kit (Gibco). DH10Bac Competent Cells (Gibco, 10361012) were transformed to generate bacmid containing FopA gene following the manufacturer’s instructions. The bacmid was analyzed with pUC/M13 primers by PCR as previously described (Jang et al., 2022) to verify the FopA encoding sequence.

### 2.3 Preparation of FopA-encoding baculovirus

ExpiSf9™ cells were transfected with a recombinant bacmid encoding the *fopA* gene for 120 hours. Following this, the recombinant baculovirus was collected through centrifugation and the isolation of supernatants. A 24-well plate containing ExpiSf9™ cells at 1.25 × 10^6^ cells/well in ExpiSf9™ CD medium was used to determine the baculovirus titer. The baculovirus was serially diluted in ExpiSf™ CD medium (1:1,000 to 1:100,000), and incubated for 14 – 16 hours at 28°C. The cells in each well were then transferred to a flow cytometry tube, and stained with an APC conjugated anti-Baculovirus envelope gp64 antibody (Thermo Fisher Scientific, 17-6991-80) for 30 minutes at room temperature. The samples were then washed, centrifuged, and analyzed using the NovoSampler Pro flow cytometer (Agilent). Data was then analyzed using NovoExpress software (Agilent), and the viral titer was calculated using the equation described below by selecting the dilution sample that yields a percent of gp64-positive cells of < 10%.


$$\text{Viral Titer}\left(\frac{\text{ivp}}{\text{mL}}\right)=\left(\;\frac{\text{Cell number}\times \text{Percent gp}64\text{ positive cells}}{\text{Dilution of virus stock}}\;\right)\times 0.01$$

### 2.4 Recombinant FopA production

Protein expression was performed with ExpiSf9™ cells at a density of 5 × 10^6^ cells/mL and ≥ 90% viability. Following seeding, ExpiSf™ Enhancer (Gibco) was added to the cells. 18-24 hours later, the cells were infected with a recombinant baculovirus stock having a MOI of 5. After 120 hours post of infection, the supernatants were harvested through centrifugation at 4,000 rpm for 30 minutes and then filtered using a 0.22-µm bottle top vaccum filter. Recombinant FopA from the filtered supernatants was purified using a cOmplete™ His-Tag Purification Column (Roche) equipped with NGC QUEST 100 Chromatography Systems (Bio-Rad). Recombinant FopA was eluted with 80mM imidazole PBS buffer and then dialyzed in PBS (pH 7.4)

### 2.5 Hybridoma generation and antibody selection

5-week-old female BALB/c were immunized with recombinant FopA antigen produced using baculovirus, and hybridomas were generated as previously described (Jang et al., 2022). The hybridomas were screened to identify mAbs against recombinant FopA by ELISA. The cDNA of hybridoma cells was synthesized using a random hexamer primer and the gene identification of the antibody VH or VL chain with PCR method, forward and reverse primer sets were designed and employed as previously described (Babrak et al., 2017). The Animal Research Ethics Committee of the Korea Research Institute of Bioscience and Biotechnology reviewed and authorized the use of animals and experimental protocols (IACUC approval no. KRIBB-AEC-21119).

### 2.6 Construction of a single chain variable fragment library

The construction of the single chain variable fragment library was carried out in three stages of PCR as previously described (Lee et al., 2018) with some modifications. Initially, the variable heavy (VH) and variable light (VL) chain sequences of the cDNA were amplified during the first PCR. In the second PCR, the flanking region containing the partial glycine and serine (G4S) linker and the SfiI enzyme site was bound to the amplified VH and VL sequences. A third PCR was performed to assemble the VH and VL thereby generating single-chain variable fragment (scFv). Each PCR product was analyzed on a 2.0% TAE agarose gel using electrophoresis. The purified gels were cleansed using the NucleoSpin^®^ Gel and PCR Clean-up kit (MACHEREY−NAGEL). The third PCR product and phagemid vector (pComb3XSS) were then digested by SfiI (NEB) at 50°C for 16 hours. The digested phagemid vector was analyzed using 0.7% agarose gel electrophoresis, and the digested vector and PCR products were purified. These products were then ligated with T4 DNA ligase (NEB) at 16°C overnight and inactivated at 65°C for 10 minutes. The ligates were then desalted using the Microcon-10kDa Centrifugal Filter Unit with Ultracel-10 membrane (Millipore). They were centrifuged at 14,000 × g for 20 minutes at room temperature, and this process was repeated two more times. The desalted and concentrated ligates were collected in a new tube by centrifuging at 3,000 × g for 3 minutes and electro-transformed into *E. coli* TG1 competent cells (Lucigen), as described previously (Lee et al., 2018). Constructed sub-libraries were titrated, mixed together, and used for phage display panning.

### 2.7 Antibody selection using phage display technology

The selection of scFv antibodies to the FopA antigen was achieved through phage display technology, as previously described (Jeon et al., 2023). Briefly, anti-FopA scFv antibodies were selected through two sets of phage display panning using immunotubes (Thermo Fisher Scientific, 444202) or Dynabeads™ M-270 Epoxy (Thermo Fisher Scientific, 14301) coated with recombinant FopA. Three rounds of bio-panning were performed, and individual scFvs binding to FopA in the third round output were screened by ELISA. To produce immunoglobulin G (IgG) antibodies, the DNA sequence of the selected scFv clones was used to clone each variable heavy and light chain genes into the pcDNA3.4-based expression vector. The anti-FopA IgG antibody was produced and purified following previously described methods (Jeon et al., 2023).

### 2.8 Indirect ELISA

Recombinant FopA protein (100 ng/well) was immobilized on a 96-well Maxisorp plate (Thermo Fisher Scientific, 439454) at 4 °C overnight. Following this, the plates were washed with PBS-T (PBS with 0.05% Tween20) and blocked with 3% (w/v) bovine serum albumin (BSA)/PBS for 1 hour 30 minutes at room temperature. For binding analysis of purified antibodies that were serially diluted in PBS, the diluted solution was added to each well, followed by incubation for 1 hour at room temperature. For hybridoma screening, supernatants of hybridoma culture media served as the primary antibody. After washing with PBS-T, a Goat anti-Human IgG F(ab’)2 - HRP (Invitrogen, 31414) was added, and incubated for 1 hour at room temperature. For supernatants of hybridoma ELISA, goat anti-Mouse IgG (Fab specific)–Peroxidase antibody (Sigma Aldrich, A9917) was used as a secondary antibody. Following another round of washing with PBS-T, tetramethylbenzidine (TMB) substrate reagent (BD Biosciences, 555214) was added to each well, and the plate was incubated. The reaction was then terminated using a stop solution, and absorbance was measured at 450 nm using a SpectraMAX ABS Plus plate reader (Molecular Devices). Supernatants collected by periplasm extraction were utilized as an alternative to the primary antibody in the ELISA assay for periplasm extraction. In this case, Anti-HA-Peroxidase (Roche, 12013819001) was employed as a secondary antibody. Curve-fitting analysis for ELISA data was performed using Prism 9 software (GraphPad Software, USA).

### 2.9 Affinity measurement using BLI

An amine-reactive second-generation (AR2G) biosensor (Sartorius) was employed to immobilize recombinant FopA, as described in prior research (Jang et al., 2022). The biosensors were treated with 1 M ethanolamine-HCl (pH 8.5) for 300 seconds to quench them, and a baseline was established using PBS for 120 seconds. The Octet K2 system (Sartorius) was used for kinetic analysis by measuring the association (K_on_) and dissociation (K_off_) of anti-FopA antibodies for 600 seconds. The equilibrium dissociation constant (KD) of each antibody was calculated using data analysis software (HT 12.0; Sartorius) based on K_on_ and K_off_.

### 2.10 Competitive ELISA

A 96-well plate coated with recombinant FopA was prepared and blocked according to the procedures outlined in the indirect ELISA method section. Subsequently, biotinylated mAbs were added to each well at a concentration of 20 nM using the EZ-Link™ Sulfo-NHS-LC-biotinylation kit (Thermo Fisher Scientific). Non-biotinylated mAbs were serially diluted from 100 nM and added to the wells along with the biotinylated mAbs. The mixture was incubated for 1 hour at room temperature, and then the wells were washed with PBST. HRP-conjugated streptavidin (Sigma Aldrich) was added to the wells and incubated for 30 minutes at room temperature. The wells were washed again, and then TMB was added and reacted as described above.

### 2.11 Sandwich ELISA

The validation of 3C1 and 6B12 mAbs was carried out using a capture mAb (50 nM) that was coated on a 96-well plate and left overnight at 4°C. Following this, the plate was blocked with 3% BSA/PBS for an hour, after which serial dilutions of recombinant FopA in 3% BSA/PBS were added to each well, and the plate was incubated for an hour at room temperature. After washing the plate, 20 nM biotinylated detector mAb was added to each well and incubated for an hour at room temperature. The plate was then washed with PBST and incubated with HRP-conjugated streptavidin (Sigma Aldrich) for 30 minutes at room temperature. This process was duplicated to assess the impact of the evaluation matrix, using bovine serum albumin (Sigma Aldrich), skimmed milk powder (Gibco), human serum (Sigma Aldrich), mouse urine, and soil water to dilute recombinant FopA at different concentrations in various matrices directly. These matrices were then transferred to 96-well plates coated with capture mAb. The detection of FopA in the other matrices was carried out as previously described.

### 2.12 Evaluation of limit of detection

The definition of LoD is the lowest concentration of FopA antigen that produces a detectable colorimetric signal, exceeding non-specific binding. For determining the LoD, linear regression analysis was executed using GraphPad Prism 9.0 (GraphPad Software, USA), and the standard deviation of the response (σ) and the slope (S) of the calibration curve were employed following the equation provided below.


LoD=3.3×σ/s

### 2.13 Statistical analysis

Data were analyzed using the means ± the standard deviation (SD) of the means, and statistical data analyses were performed by GraphPad Prism 9.0 (GraphPad Software, USA).

## 3 Results

### 3.1 Recombinant FopA antigen production and mouse immunization

**Figure 1:**
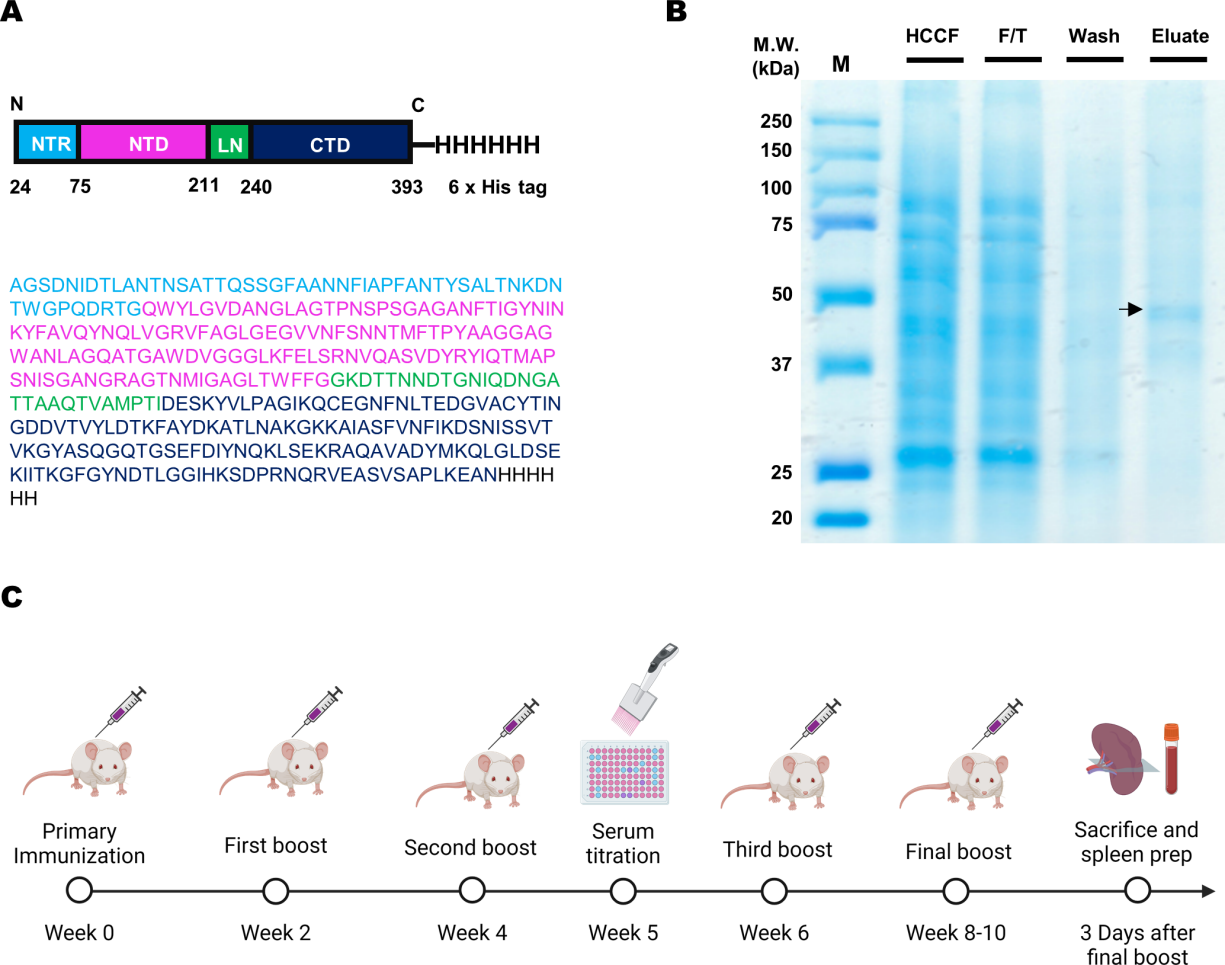
Recombinant FopA protein expression and mouse immunization. **(A)** Schematic illustration of the expressed domains of FopA protein with polyhistidine tags and amino acid sequence of FopA. **(B)** The production of recombinant FopA protein was analyzed using SDS-PAGE. The harvested cell culture fluid (Lane 1), flow-through fraction (Lane 2), washed fraction (Lane 3), and elution fraction (Lane 4). **(C)** Schematic representation of the mouse immunization protocol. The mice were immunized four to five times at 2-week intervals, and serum was collected after the second boost step for serum antibody titration. The spleen was collected 3 days after the final boost. Created with BioRender.com.

To produce the FopA antigen and generate FopA antibodies, we utilized a Baculovirus-insect cell expression vector system (Jang et al., 2022). The *fopA* gene containing the 6 × His tag was inserted into the pFastBac donor plasmid, transformed into competent cells, and bacmid DNA was isolated and confirmed by agarose gel electrophoresis (**Figure 1A**; **Supplementary Figures 1A, B**). Pure recombinant FopA protein, corresponding to approximately 44 kDa, was purified using a Ni-NTA column and analyzed by SDS-PAGE and Coomassie Brilliant Blue staining (**Figure 1B**). BALB/c mice were immunized with recombinant FopA for hybridoma and phage display screening using a single-chain variable fragment (scFv) library. Initially, 5-week-old BALB/c mice were immunized with the antigen with complete Freund’s adjuvant by intraperitoneal injection, followed by three subcutaneous injections of the antigen with incomplete Freund’s adjuvant fortnightly for boost immunization. The final booster immunization was performed by intravenous injection to increase the intensity of the booster (**Figure 1C**).

### 3.2 Screening of antibodies binding to FopA antigen by immune library and hybridoma

**Figure 2:**
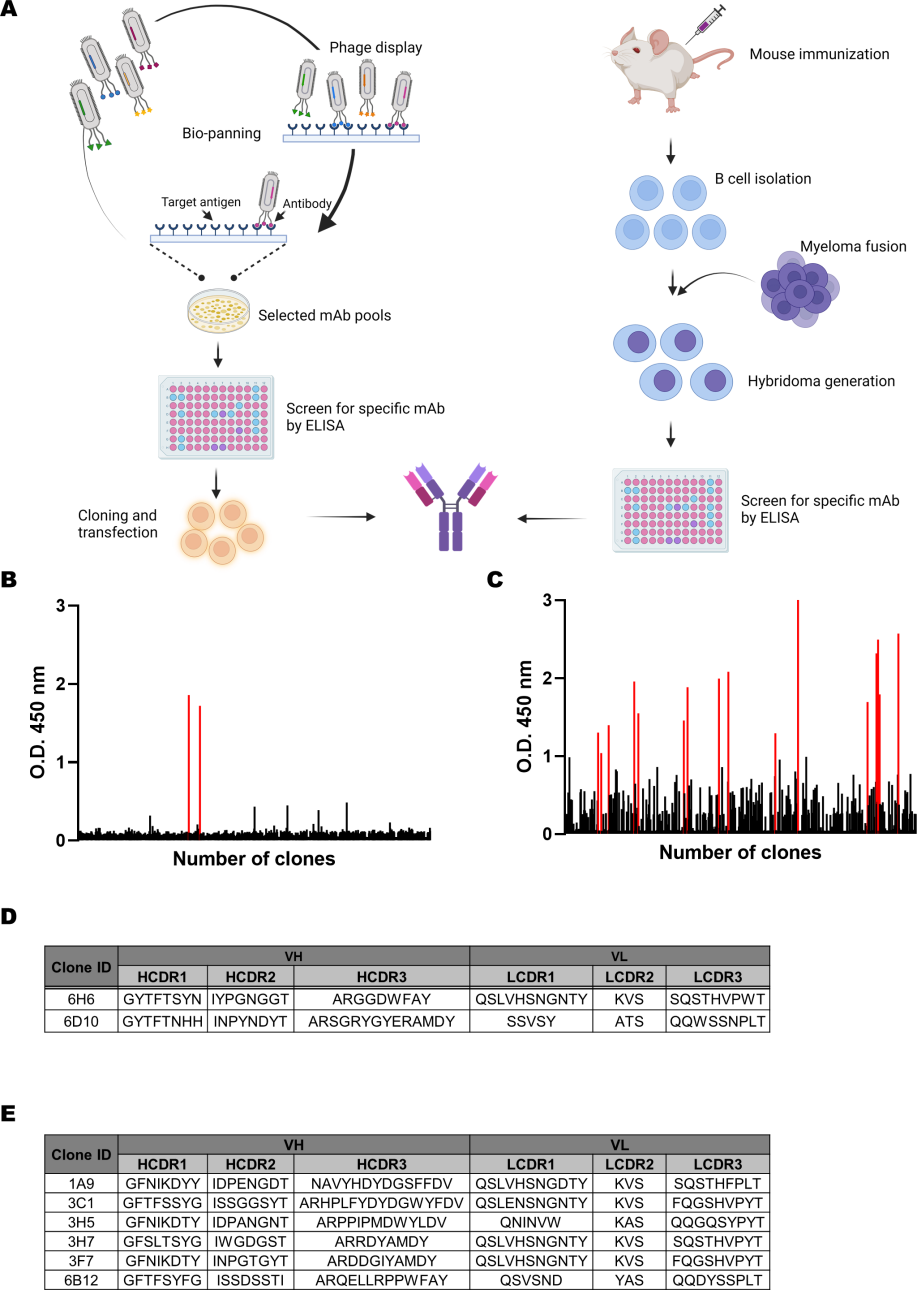
Antibody screening with Immune library and Hybridoma technology. **(A)** Schematic representation of the Hybridoma antibody screening method and phage display screening method with immune antibody library. **(B, C)** Screening of antibodies against the FopA recombinant protein. Out of the 954 antibody clones produced from hybridoma screening, 6H6 and 6D10 (O.D. 450nm > 1.0) were selected. 16 positive clones (O.D. 450nm > 1.0) were selected from the total of 672 scFv clones produced by the immune library. **(D, E)** Sequence identification of the variable heavy (VH) and light (VL) chains of anti-FopA antibodies. The VH and VL regions of each antibody were sequenced to identify the complementarity-determining regions (CDRs). Created with BioRender.com.

To select highly specific and diverse antibodies that bind to FopA, we used the hybridoma method and phage display in conjunction with an immune library (**Figure 2A**). This involved fusing Sp2/0 myeloma cells and splenocytes isolated from FopA-immunized mice to generate and screen hybridoma clones based on their binding activity to recombinant FopA using ELISA. After screening, two candidate mAbs were selected for DNA sequencing (**Figure 2B**). To construct an immune scFv library against FopA, total RNA was isolated from the splenocytes of immunized mice and cDNA was synthesized via reverse transcription PCR. The VH and VL regions were amplified using multiple primers to generate the scFv genes (**Supplementary Figure 2**). Biopanning was performed three times using the FopA antigen, and polyclonal antibodies from each round were expressed by IPTG induction (**Supplementary Figures 3A, B**). The antigen-binding activity of these antibodies was evaluated by ELISA. The binding of polyclonal anti-FopA antibodies from the third-round output was significantly greater than that observed in previous rounds or in the initial immune library (**Supplementary Figures 3C, D**). This suggests that the panning rounds successfully amplified the target-binding clones. To further analyze the output of the third round, we screened individual scFv clones and identified 16 positive binders for the antigen (**Figure 2C**). By analyzing DNA sequences of anti-FopA antibodies, we finally determined the complementarity-determining regions (CDRs) in the variable heavy (VH) and variable light (VL) chain sequences of two (**Figure 2D**) and six (**Figure 2E**) antibodies derived from hybridomas and phage display panning, respectively.

### 3.3 Production and characterization of monoclonal antibodies against FopA

**Figure 3:**
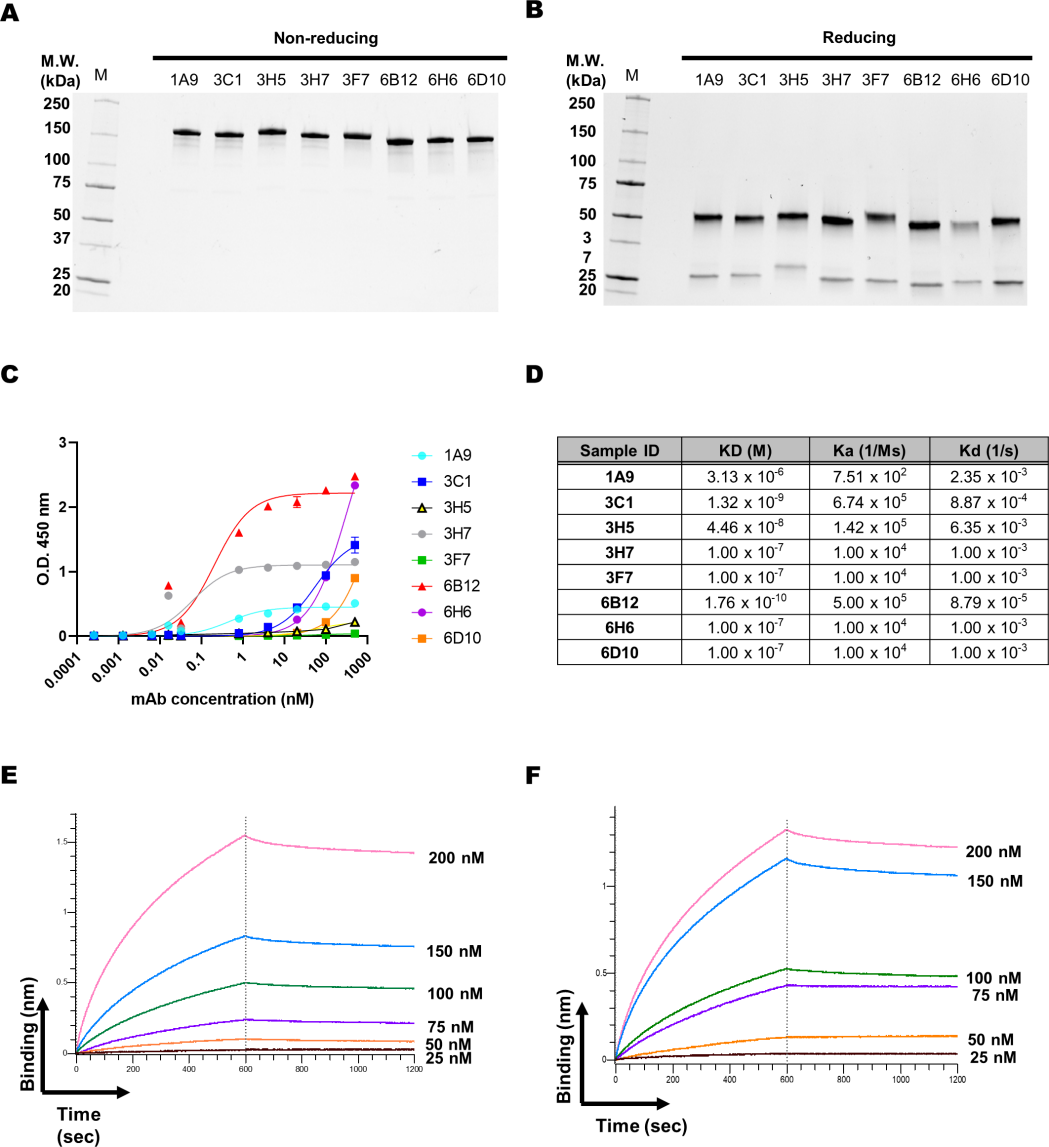
Antibody production and characterization. Two IgG antibodies were purified from hybridoma and six IgG antibodies from an immune library. The expressed antibodies were purified using a protein A column and analyzed by SDS-PAGE to determine their sizes and purity. **(A)** The non-reducing condition showed an intact size of approximately 150 kDa, while **(B)** the reducing condition showed sizes of 50 kDa (Heavy chain) and 25 kDa (Light chain). **(C)** The binding activity of the eight IgG antibodies was validated using recombinant FopA protein. ELISA assessed the dose-dependent binding of 6B12, 3H7, and 3C1 IgGs. **(D)** The binding affinity of the eight antibodies was measured using biolayer interferometry. Recombinant FopA was immobilized on an AR2G biosensor and allowed to bind to the antibodies. **(E, F)** For detailed analysis, the antibodies were immobilized on an AR2G biosensor and subsequently permitted to bind with the FopA antigen diluted to various concentrations (25, 50, 75, 100, 150, and 200 nM). Kinetic rates and equilibrium binding constants were analyzed using global fitting analysis of the binding curves. Values represent the mean ± SD for a duplicate **(C)**.

To evaluate the binding affinity of the antibodies, scFvs obtained from the immune library were converted into IgG, transfected into Expi293F cells, transiently expressed, and purified for measurement (Jeon et al., 2023). In contrast, 6H6 and 6D10 hybridoma clones were not produced as an intact IgG form (data not shown). Therefore, the VH and VL chains of these clones were individually cloned into heavy chain (HC) and light chain (LC) expression vectors containing the constant region. HC and LC expression vectors were generated for each antibody and were transiently expressed in Expi293F cells. The antibodies were verified under both non-reducing and reducing conditions and their size and purity were determined using SDS-PAGE (**Figures 3A, B**). The binding activity of IgGs was initially validated by ELISA, which demonstrated that 6B12, 3H7, and 3C1 clones specifically bound to FopA in a dose-dependent manner (**Figure 3C**). Biolayer interferometry (BLI) was used to determine the affinity of these antibodies. Recombinant FopA was immobilized on a biosensor tip and 100 nM of each antibody was allowed to bind to the antigen (**Figure 3D**). Our results revealed that the anti-FopA 6B12 and 3C1 antibodies exhibited the highest binding affinity among the tested antibodies, with apparent K_D_ of 1.76 × 10^-10^ M and 1.32 × 10^-9^ M, respectively. Therefore, these two antibodies were selected for further analyses. For detailed examination, either 6B12 or 3C1 IgG was immobilized on a biosensor tip and subjected to varying concentrations of the FopA antigen. The binding affinities were calculated for their association and dissociation values at six different concentrations of antigen, and the K_D_ value of 6B12 was 5.06 × 10^-9^ M, and 3C1 was 5.62 × 10^-9^ M (**Figures 3E, F**). These findings demonstrate that anti-FopA 6B12 and 3C1 antibodies are produced purely in IgG form and have a prominent binding affinity for a specific antigen, making them promising candidates for immunoassays.

### 3.4 Development of a sandwich ELISA method for the detection of FopA

It is vital to ensure that a suitable combination of antibodies is selected for the development of a sandwich ELISA-based detection system, considering that different antibody sequences may possess distinct epitopes on the target (Jang et al., 2022). To this end, we conducted a competitive binding assay to evaluate the efficacy of biotinylated and non-biotinylated antibodies in detecting the FopA antigen.

**Figure 4:**
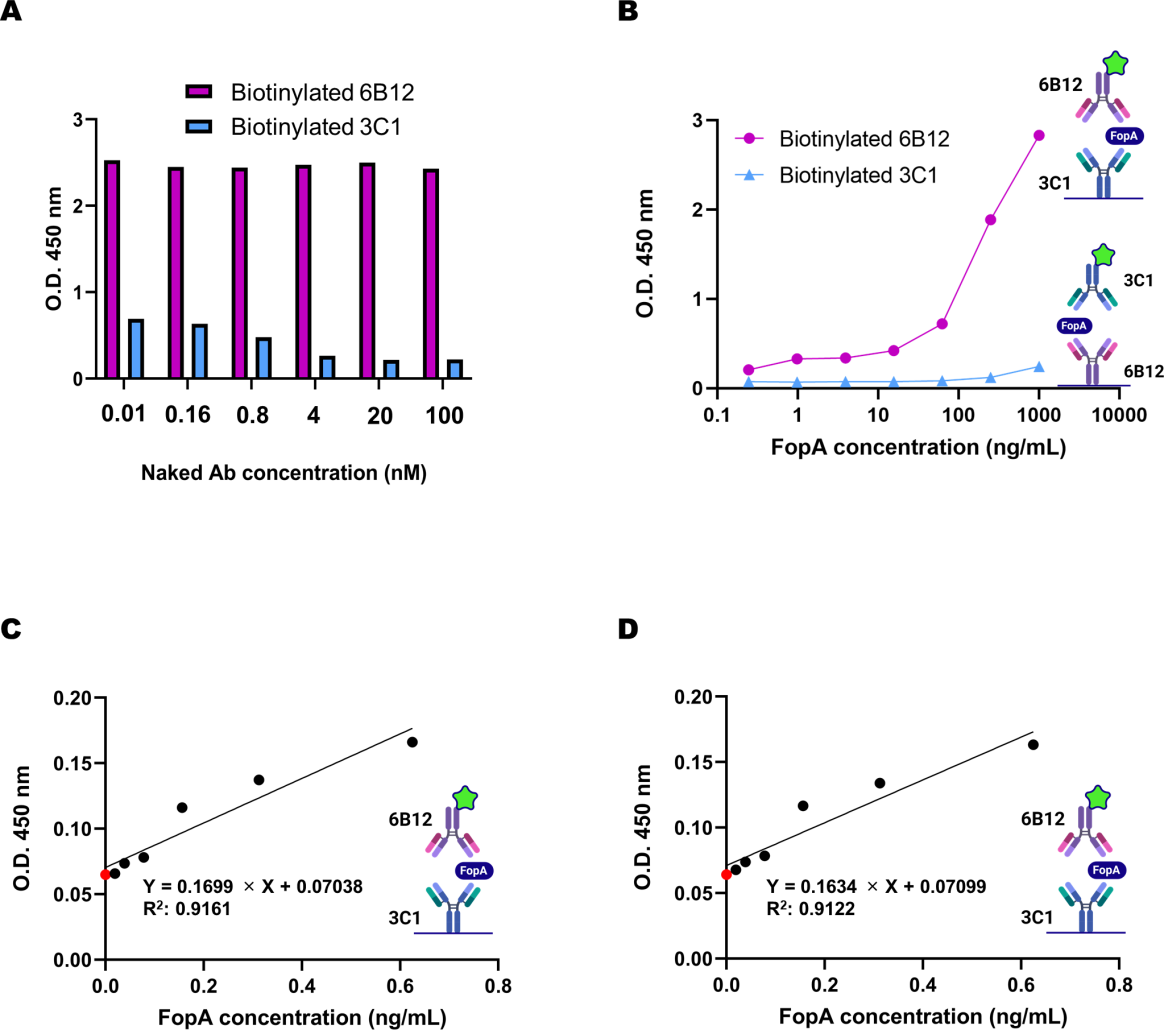
Development of a method for detecting FopA antigen using a sandwich ELISA. **(A)** Two combinations of antibodies were evaluated for competitive ELISA using recombinant FopA. Each biotinylated antibody was fixed at 20 nM concentration, and each naked antibody diluted one-fifth serially from 100 nM concentration was incubated with the antigen. **(B)** The detection efficiency of the two combinations of antibodies, 3C1 (capture) and biotinylated 6B12 (detector), or biotinylated 3C1 (detector) and 6B12 (capture), was assessed using the sandwich ELISA method. **(C, D)** The limit of detection was determined by incubating each capture mAb (50 nM) coated on a 96-well ELISA plate and using a biotinylated detector mAb (20 nM) to detect 0.062 ng/mL of FopA in PBS, 0.064 ng/mL in 3% skim milk, and a red dot indicating the background signal. The limit of detection value was calculated as described method section. Values represent the mean ± SD for a duplicate **(A)** and a triplicate **(B–D)**. Created with BioRender.com.

As depicted in **Figure 4A**, our results demonstrated that, regardless of the concentration of the naked 3C1 antibody, the biotinylated 6B12 antibody was able to bind to the antigen. In contrast, when a biotinylated 3C1 antibody was used, its binding to the antigen was hindered by the naked 6B12 antibody. These findings were confirmed using sandwich ELISA, in which the 3C1 antibody was used as the capture antibody, and the 6B12 antibody was used as the detector antibody. As the concentration of the FopA antigen increased, so did the absorbance, indicating that 6B12 is a suitable candidate as a detector antibody, whereas 3C1 is suitable as a capture antibody in a sandwich ELISA configuration (**Figure 4B**). Furthermore, analysis of the limit of detection (LoD) demonstrated that this pair of antibodies was highly specific and sensitive, exhibiting detection limits of 0.062 ng/mL in PBS and 0.064 ng/mL in skim milk matrices (**Figures 4C, D**).

### 3.5 Validation of sandwich ELISA for detecting FopA in diverse matrices

**Figure 5:**
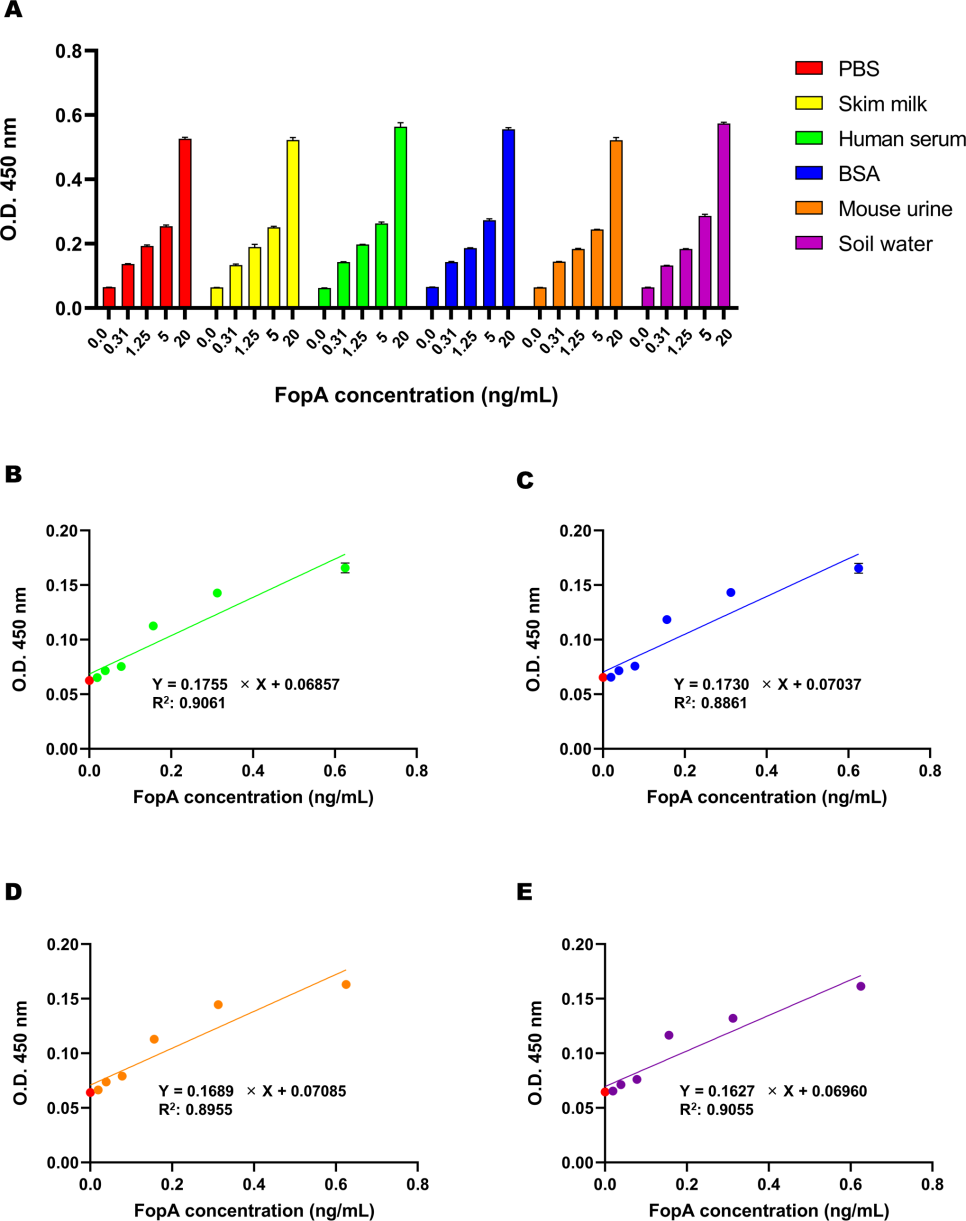
Detection of FopA using the sandwich ELISA in various matrices. The evaluation of a pair of antibodies for detecting recombinant FopA using sandwich ELISA in various matrices, including 3% human serum, 3% bovine serum albumin (BSA), mouse urine, and soil water. **(A)** The capture monoclonal antibody (50 nM) coated on a 96-well ELISA plate and biotinylated detector monoclonal antibody (20 nM) were employed to assess the FopA concentration serially diluted from 20 ng/mL. The detection sensitivity of FopA was evaluated by serial dilution of the sample, resulting in a determined limit of detection (LoD) of 0.066 ng/mL for 3% human serum **(B)**, 0.074 ng/mL for 3% BSA **(C)**, 0.071 ng/mL for mouse urine **(D)**, and 0.067 ng/mL for soil water **(E)**, and a red dot indicating the background signal. Error bars represent standard deviations from a triplicate.

We evaluated the potential of our immunoassay method to diagnose *F. turalensis* in clinical and contaminated samples using FopA protein in various matrices. As shown in **Figure 5A**, recombinant FopA was successfully detected at a range of 0.3–20 ng/mL when diluted in PBS, skim milk, human serum, bovine serum albumin (BSA), mouse urine, and soil water. Our results indicated that recombinant FopA protein could be sensitively detected at an approximate 0.3 ng/mL concentration across all matrices. To the impact of various matrices on the detection of FopA protein, we conducted linear regression analyses and found that human serum, BSA, mouse urine, and soil water had little to no effect on the LoD values, which were determined in the range of 0.066 to 0.074 ng/mL (**Figures 5B–E**). Therefore, an immunoassay method utilizing anti-FopA monoclonal antibodies is capable of detecting FopA without interference from different matrices, including human sera, and effectively identifying pathogens in contaminated samples across various environments.

## 4 Discussion

Given its status as a potentially infectious disease, it is essential to promptly and accurately diagnose tularemia, a disease with a range of clinical symptoms and potentially fatal consequences. This is particularly crucial in situations of widespread exposure, such as during a pandemic, where quick and accurate diagnosis can save lives (Hannah et al., 2021). To provide a more precise and prompt diagnosis of *F. tularensis*, we developed a sandwich ELISA test that employs a novel antibody designed to bind FopA, a distinctive outer membrane protein antigen of *F. tularensis*. While most existing antibody-based immunoassays target the LPS found in *F. tularensis*, this method raises concerns regarding false-positive results. We developed a novel diagnostic method that has not been previously reported, utilizing antibodies that specifically bind to FopA. The protein used in our new diagnostic method is a member of the OmpA family and exhibits low sequence homology with that of other gram-negative bacteria (Nagaratnam et al., 2022). Moreover, FopA has a high copy number on the outer membrane of bacteria and is immunogenic, making it a suitable target for antibody development (Confer and Ayalew, 2013). Previous studies have suggested that monoclonal antibodies against FopA can inhibit *F. tularensis* pathogenicity. However, they have not been used for diagnosis or to provide detailed information on their sensitivity and specificity (Savitt et al., 2009).

Immunoassays are capable of providing quick and precise diagnoses compared to PCR based-or cell culture-based diagnostic methods (Liu et al., 2021). To achieve optimal results using immunoassays, it is important to develop antibodies with high specificity and sensitivity (Cox et al., 2019). To this end, we utilized an insect cell based expression system to produce high-purity recombinant FopA proteins for the development of high-quality antibodies, and we employed two different methods for generating diagnostic antibodies of diverse sequences: the mouse-derived immune library screening method and the hybridoma screening method. The insect expression system has proven to be advantageous for antigen preparation, as it ensures a sufficient amount of protein while minimizing the likelihood of endotoxin-induced immunogenicity, which can occur when proteins are produced by microorganisms (Mamat et al., 2013; Tripathi and Shrivastava, 2019). In this study, we employed two methods for antibody screening: a hybridoma screening system, and an immune library screening method using phage displays. Although the hybridoma screening system is an effective method for developing high-affinity antibodies through natural affinity maturation, it has the disadvantage of low efficiency in cell fusion and hybridoma isolation, requiring considerable time to generate a cell line and select a specific hybridoma. Moreover, hybridoma cell lines can be genetically unstable and their cultures are at constant risk of contamination (Moraes et al., 2021). In contrast, the use of phage displays for immune library screening has proven to be a feasible approach for identifying a broad range of high-affinity antibodies. This method offers significant advantages, specifically by enabling the selection of numerous sequences during the panning process and facilitating the rapid isolation and characterization of monoclonal antibodies with high specificity. Additionally, it allows for easy modification of diverse antibody formats, making it a versatile tool for antibody discovery (Clementi et al., 2012; Moraes et al., 2021). Unfortunately, we encountered difficulties generating diagnostic-grade antibodies during the hybridoma screening process. The clones derived from the hybridoma process faced challenges in expressing a form of IgG antibodies, and even when additional cloning processes were applied, the resulting antibodies had a low affinity compared to those selected from the immune library.

We validated two antibody candidates that recognized distinct epitopes of FopA and can be utilized in a sandwich ELISA diagnosis test for *F. tularensis*. Therefore, it is essential to evaluate the diagnostic potential of these antibodies in various matrices to confirm their effectiveness. This sandwich ELISA method demonstrated exceptional performance in multiple matrices, including human sera, with detection limits of 0.062–0.074 ng/mL. This diagnostic method maintains high sensitivity and specificity regardless of sample contamination. Commercial immunodiagnostic kits evaluate *F. tularensis* infections by detecting IgG or IgM antibodies specific to the LPS antigen of the bacterium (Yanes et al., 2018). These methods are commonly used to identify antibodies in the blood of patients suspected of infection (Maurin, 2020). However, these diagnostic methods may result in false-positive outcomes due to the presence of anti-LPS antibodies from other gram-negative bacteria (Sharma et al., 2013). Furthermore, the diagnosis of infection before *F. tularensis* antibody generation can be difficult because of the limitations of these methods (Maurin, 2020). Additionally, when using anti-LPS detection antibodies, the possibility of false-positive results for other infectious bacteria and the low sensitivity of these antibodies when detecting infections in patient sera should be taken into account (Hannah et al., 2021). We have not been able to directly detect *F. tularensis* using the actual pathogen owing to its unavailability in Korea. However, our study suggests that novel anti-FopA antibodies could serve as a promising alternative for diagnosing *F. tularensis* infections, offering high sensitivity and specificity without inducing cross-reactivity.

It is important to identify highly specific antigens and acquire diverse antibody candidates to improve the sensitivity of early pathogen detection. Hybridoma technology is a highly effective antibody-screening method; however, it presents difficulties for high-throughput screening. Incorporating a technique that isolates single B cells to identify high-affinity antibodies against the antigen following mouse immunization could be a potential solution (Pedrioli and Oxenius, 2021). Additionally, the immune library screening method employs NGS-based antibody sequencing to obtain diverse sequences (Yang et al., 2017).

## 5 Conclusion

In conclusion, we have successfully developed a range of antibodies that specifically bind to FopA, an outer membrane protein of *F. tularensis*, a pathogen that poses a significant infective risk. These antibodies can be used for early detection and diagnosis of infections caused by this pathogen. Prompt diagnosis is crucial for controlling the rapid spread of infectious diseases and preventing instances of bioterrorism. Existing antibody-based diagnostic systems for *F. tularensis* rely on targeting LPS proteins; however, our system utilizes antibodies against FopA as a novel diagnosable antigen. The development of these diagnostic antibodies highlights the potential of immunodiagnostics based on antibodies targeting other outer membrane proteins, as well as those targeting LPS, for diagnosing pathogens. This finding could serve as a basis for future research exploring the therapeutic use of antibodies that bind to FopA in humans infected with *F. tularensis*.

## Data Availability

The original contributions presented in the study are included in the article/**Supplementary Material**. Further inquiries can be directed to the corresponding authors.
