# Supplementary material for: Development of a novel sandwich immunoassay based on targeting recombinant Francisella outer membrane protein A for the diagnosis of tularemia
Source: Front Cell Infect Microbiol. 2024 Aug 20;14:1455259. doi: 10.3389/fcimb.2024.1455259 (PMC11368854; doi:10.3389/fcimb.2024.1455259)
Supplement: Supplementary file 1 [file DataSheet1.docx]

Supplementary Material

Development of a novel sandwich immunoassay based on targeting recombinant Francisella outer membrane protein A for the diagnosis of tularemia

Jieun Jang, Do Hyung Kwon, Ju-Hong Jang, Dong-Gwang Lee, Seo-Hyuk Chang, Min-Young Jeon, Young-Su Jeong, Dong-Hyun Song, Jeong-Ki Min, Jong-Gil Park, Moo-Seung Lee, Baek-Soo Han, Wonjun Yang*, Nam-Kyung Lee*, Jangwook Lee*

*** Correspondence:**

Corresponding author’s E-mail address

wonjun@kribb.re.kr, nklee@kribb.re.kr, and jlee@kribb.re.kr

# Supplementary Figures and Tables

## Supplementary Figures


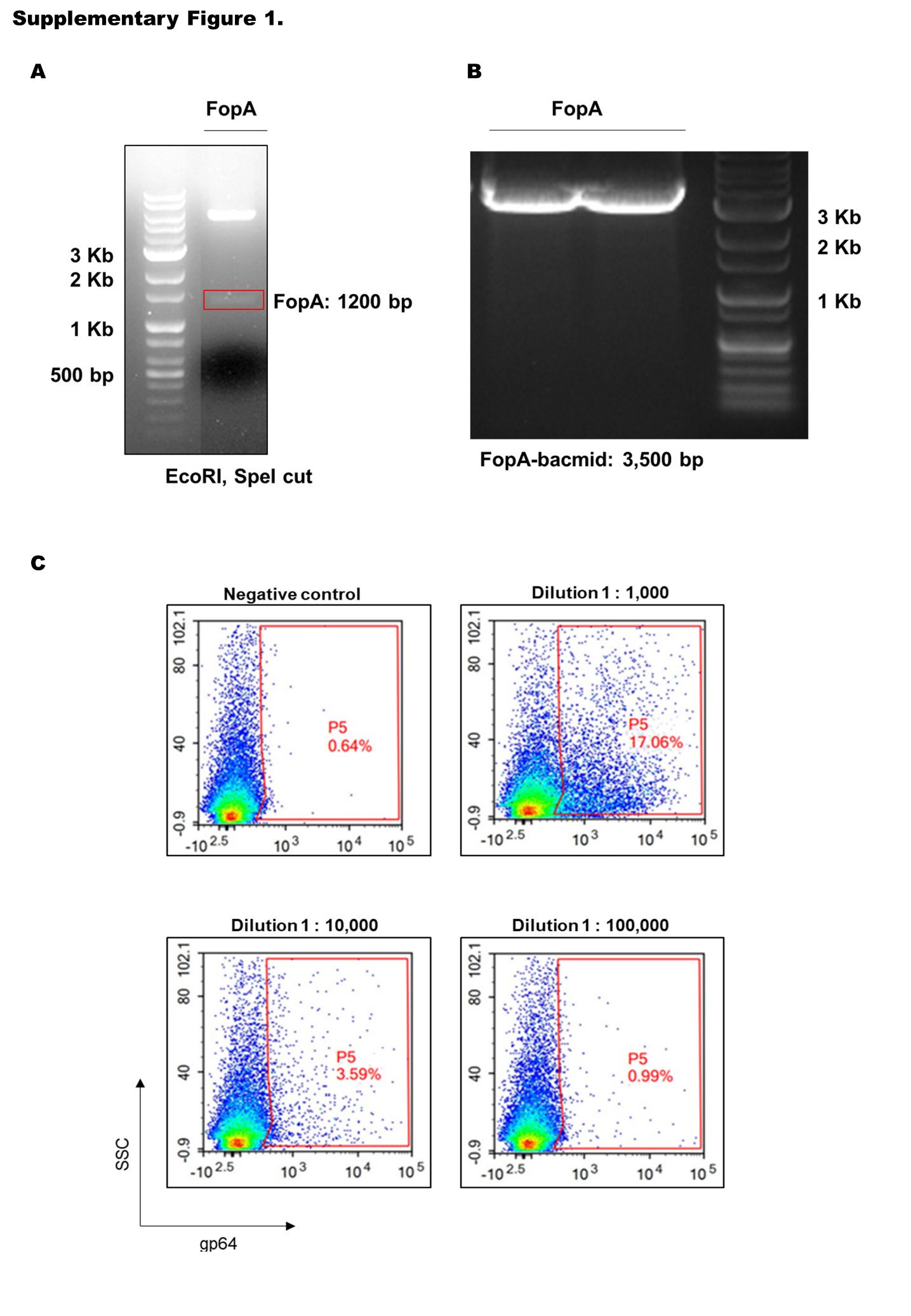


**Supplementary figure 1.** **Generation of FopA bacmid and encoding baculovirus.**

(A) The pFastBac vector was digested with the EcoRI and SpeI restriction enzymes. By agarose gel electrophoresis, it was confirmed that approximately 4,700 base pairs of the pFastBac vector and 1,200 base pairs of the FopA (red box) gene. (B) The bacmid containing the FopA gene was also analyzed by agarose gel electrophoresis, revealing that about 3,500 base pairs of amplified bacmid containing FopA gene were confirmed. (C) The FopA-encoding baculovirus was packaged from insect cells and serially diluted before being introduced to Sf9 cells. The expression level of the surface marker gp64 for baculoviral infection was measured as a percentage of the gate of non-infected cells (negative control).


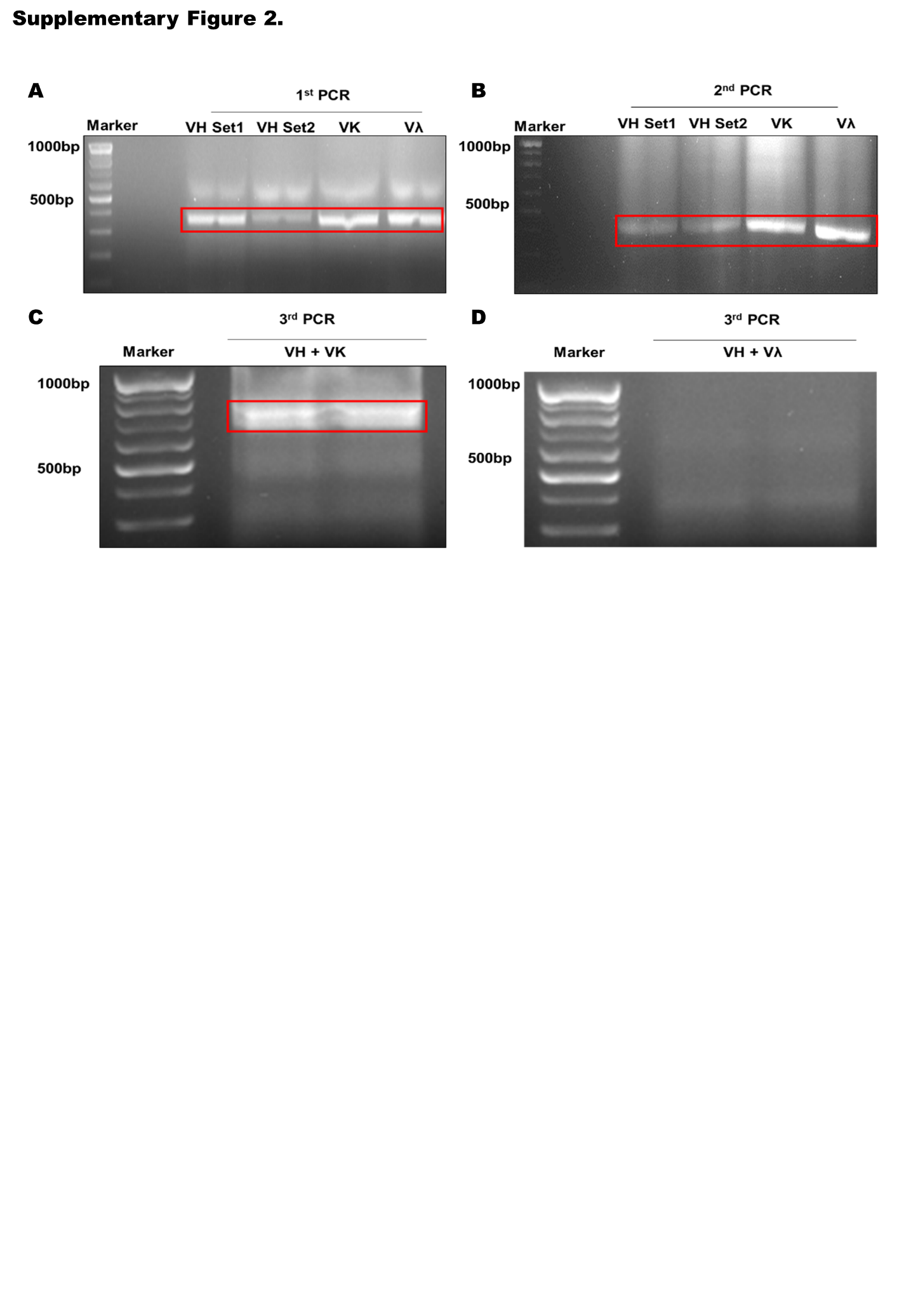


**Supplementary figure 2. Mouse immune libraries amplified VH and VL regions of splenocytes.**

(A) The initial PCR amplified the VH and VL regions, the product represented approximately 400 base pairs. (B) A second PCR was performed to add an incomplete linker and a SfiI enzyme site to the VH and VL regions. The resulting product was again about 400 base pairs. (C) A third PCR was carried out to link the VH and VL regions. The resulting fragment, approximately 800 base pairs in size (red box on the left), indicates successful linking. However, (D) the VH and Vλ regions remained unamplified (right).


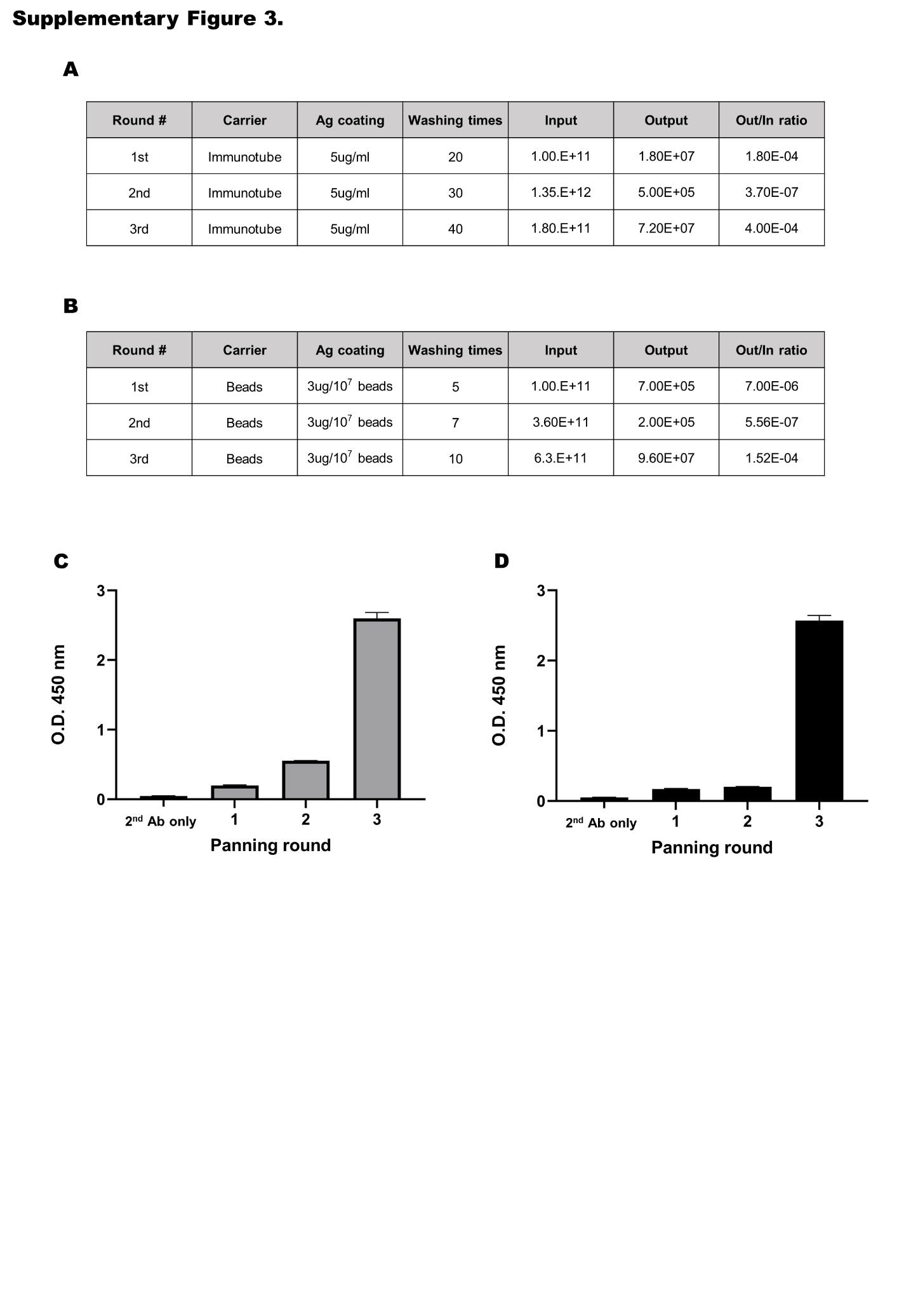


**Supplementary figure 3. The result of bio-panning and polyclonal antibody binding titer.**

Bio-panning was carried out in two different conditions, with recombinant FopA coated on (A) immunotube at a concentration of 5 µg/ml and (B) 3 µg/107 epoxy beads. The number of washing times varied between 20 and 30 in the case of immunotube, and between 5, 7, and 10 in the case of epoxy beads (A, B). After the 3rd round of panning, polyclonal scFv antibodies were expressed by IPTG induction and used to evaluate their binding activity to FopA using ELISA (C) Immunotube, (D) Beads. Values represent the mean ± SD for a duplicate.
